# A double-blind randomised controlled trial protocol comparing dexamphetamine and placebo effectiveness for obesity management in primary care settings

**DOI:** 10.1016/j.mex.2026.103944

**Published:** 2026-05-04

**Authors:** Natalie Gauci, Emily Hibbert, Hazer Khalifa, Alison Poulton

**Affiliations:** a Nepean Clinical School, The University of Sydney, Sydney Australia; b Nepean Lung and Sleep, Kingswood Australia; c Charles Perkins Centre, The University of Sydney, Sydney Australia; d Nepean Hospital, Kingswood Australia

**Keywords:** Dexamphetamine, Obesity, Weight loss, Anti-obesity medication

## Abstract

Obesity is a growing health and economic burden. This study investigates dexamphetamine, an inexpensive drug, as a potential safe and effective treatment for obesity when combined with diet and lifestyle changes over six months in general practice. The trial is a double-blind, randomised, placebo-controlled study with a six-month active treatment phase and two years of follow-up. Participants are randomised 1:1 to receive either dexamphetamine or placebo, starting at 5 mg twice daily, with weekly titration up to 30 mg twice daily as tolerated. Weekly reviews during titration assess tolerability and support lifestyle modifications, followed by monthly reviews during the maintenance phase. Dose adjustments are guided by clinical effects and a standardised amphetamine rating scale, with gradual down-titration during the final month. If demonstrated to be effective and safe long-term, dexamphetamine could provide a scalable, practical solution to reduce the individual and societal burden of obesity.

## Graphical abstract

**Figure:**
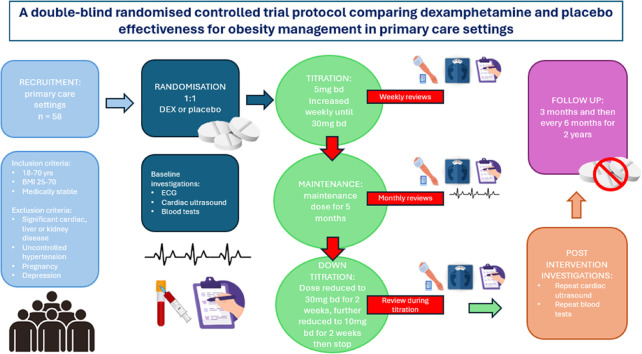

## Specifications table

**Table:** 

**Subject area**	Medicine and Dentistry
**More specific subject area**	Obesity management
**Name of your protocol**	A double-blind randomised controlled trial protocol comparing dexamphetamine and placebo effectiveness for obesity management in primary care settings
**Reagents/tools**	Aspen Dexamphetamine sulfate 5 mg tablet
**Experimental design**	This is a double-blind randomised placebo-controlled trial of a 6 month active treatment period followed by 2 years of observation. Participants are randomised 1:1 to either immediate release dexamphetamine 5 mg tablets or placebo tablets. The dose starts at 1 tablet twice daily and is up titrated weekly by 1 tablet twice daily as tolerated, to a maximum of 6 tablets (30 mg) twice daily. Participants are reviewed weekly during titration to assess medication tolerability and to reinforce positive lifestyle behaviours, followed by monthly reviews at maintenance dose. Dosage adjustment is guided by clinical effects, and a standardised rating scale for amphetamine effects. The dose is down titrated over the last month of the treatment period and then ceased.
**Trial registration**	Australian and New Zealand Clinical Trials Registry (ANZCTR) ACTRN12625000764437p
**Ethics**	This study involves human participants, and informed consent will be obtained from all participants prior to their inclusion in the study, in accordance with ethical standards.
**Value of the Protocol**	• This research will provide valuable insights into how DEX can be integrated into current obesity management protocols
	• Assess the implementation, safety and feasibility of using DEX to treat obesity in general practice settings
	• DEX offers a promising alternative that balances efficacy, affordability, and accessibility

## Background

This study investigates the potential of dexamphetamine (DEX) as a novel, cost-effective treatment for obesity, using individualised dose titration over a 6-month period. Drawing on extensive clinical experience with DEX in the treatment of attention deficit hyperactivity disorder (ADHD), this trial aims to repurpose a well-established and relatively safe medication to support weight loss, alongside sustainable dietary and lifestyle modifications [1]. If successful, the study may define a therapeutic dose range for obesity and demonstrate that DEX, when combined with behavioural support, can achieve clinically meaningful outcomes.

Compared to other Therapeutic Goods Australia (TGA) approved anti-obesity pharmacotherapies, which are often prohibitively expensive, DEX represents a more affordable alternative. This is particularly relevant for individuals from socioeconomically disadvantaged backgrounds, who experience higher rates of obesity and have limited access to treatment options [2]. Broadening access to effective pharmacotherapy could, therefore, have a substantial positive impact on public health. However, proper titration protocols and safety measures must be firmly established before wider clinical use.

For implementation in general practice, several key components are required. First, general practitioners (GPs) must receive comprehensive education on DEX titration principles, patient monitoring, and safety protocols. Clinical guidelines will be essential to assist GPs in identifying appropriate patients, including those with minimal cardiovascular or psychiatric risk factors. Integration of care through collaboration between GPs, dieticians, and mental health professionals will ensure a holistic approach that addresses both the pharmacological and behavioural aspects of obesity management.

Safety remains a primary consideration. Although DEX is approved for long-term use in children as young as six with ADHD and increasingly prescribed to adults in the form of lisdexamfetamine, prescribing regulations vary across Australian states [3]. In some regions, GPs are authorised to prescribe stimulants, while others impose restrictions. Advocacy for uniform national policies would help ensure equitable access and consistent standards of care.

The outcomes of this study could pave the way for larger trials evaluating DEX as a time-limited intervention for adults with overweight or obesity. If long-term efficacy and safety are demonstrated, DEX could represent a scalable, practical solution that reduces the burden of obesity both at the individual and societal levels. Ultimately, integrating DEX into routine care could expand the treatment toolkit available to GPs and improve outcomes for a population in critical need of effective intervention.

## Description of protocol

### Study aim

The primary aim of this study is to evaluate the implementation, safety, and feasibility of DEX as a treatment for obesity in general practice settings. The study will assess how DEX can be incorporated into routine primary care and its potential role as an accessible and affordable therapeutic option for obesity management.

### Study objectives

#### Primary objective

To evaluate the effect of DEX treatment on body weight in patients with obesity managed in general practice.

Secondary Objectives

1. To assess the cardiovascular safety of DEX treatment through monitoring of cardiovascular parameters and the occurrence of adverse cardiovascular events.
2. To identify possible undiagnosed active depressive disorders at baseline prior to initiation of treatment.
3. To evaluate changes in mood, cognition, and physiological responses during treatment using the Amphetamine Interview Rating Scale (AIRS).
4. To assess the feasibility of implementing DEX therapy for obesity management within routine general practice care, as measured by recruitment, retention, adherence, and tolerability.

### Design overview

This study is a randomised, double-blind, two-group clinical trial conducted in a general practice setting. Eligible participants will undergo a screening and baseline assessment before being randomised in a 1:1 ratio to receive either the active medication (DEX) or a placebo, with both groups receiving identical tablets. All participants will receive exercise and dietary advice from a dietician at baseline and again at the end of the 6-month treatment phase.

**Fig. 1:**
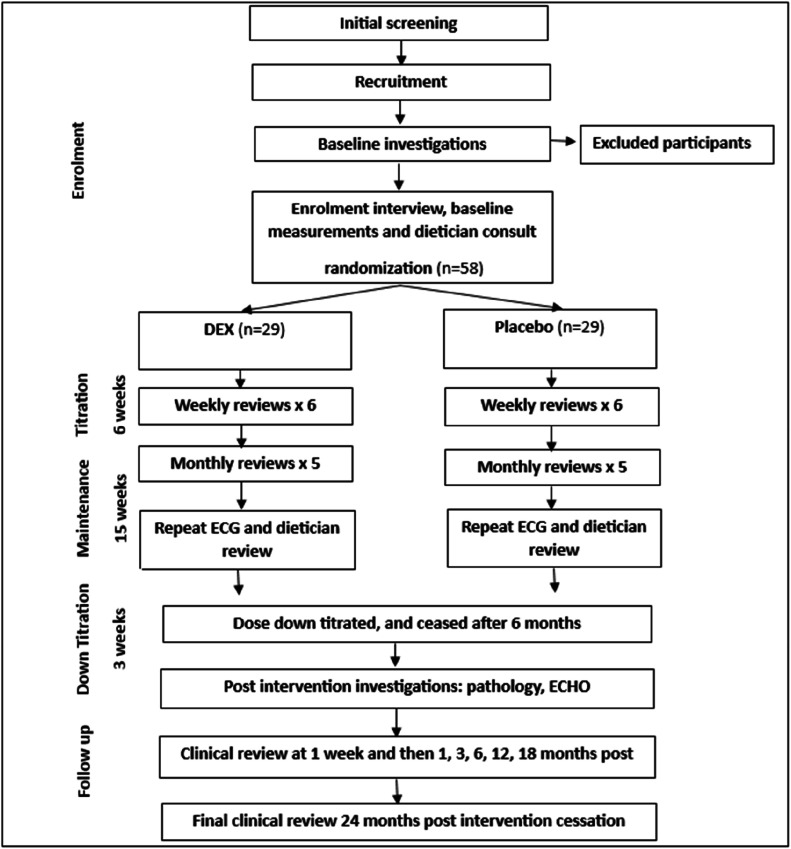
CONSORT Diagram of study protocol. This figure outlines the flow of participants through each stage of the study, from recruitment to follow-up.

During the initial 6-week dosage titration period, participants will be reviewed weekly by a clinician, followed by monthly reviews for the remainder of the 6-month treatment period and down titration. Medication will be supplied monthly to each participant. Upon completing the treatment period, participants will be reviewed one week after discontinuing their assigned study medication, with follow-up visits scheduled at one, three, and six months, and continuing every six months for a total of two years (Fig. 1).

Dose adjustments will be made based on clinical indications, with input from both the clinician and the participant. Several parameters will guide these adjustments, including: 1) weight, blood pressure, and heart rate, 2) amphetamine effects, assessed using the Amphetamine Interview Rating Scale (AIRS), 3) stated adherence to the prescribed diet and exercise program.

### Participants

Potential participants will be screened to determine whether they meet inclusion criteria and any exclusion criteria prior to recruitment.

Inclusion Criteria:

a) Adults aged 18–70 years
b) BMI 25–70
c) Medically stable, defined as no acute medical illness within the past 3 months, no uncontrolled chronic disease, and no recent changes in medications within past 3 months.

Exclusion Criteria:

a) Significant cardiac valve disease, cardiomyopathy, or arrhythmia
b) Symptomatic ischemic heart disease
c) History of addiction to illicit drugs
d) Uncontrolled hypertension >140/90
e) Significant kidney or liver disease
f) Uncontrolled epilepsy
g) Breastfeeding, pregnant, or planning pregnancy
h) Current depression or other psychiatric illness
i) Current or recent treatment (past 12 months) with psychotropic medication, systemic glucocorticoids, or weight loss medication (e.g., orlistat, phentermine)
j) Family history of sudden death from cardiac causes
k) Hypersensitivity to DEX or any components of the DEX or placebo tablet

All participants will be fully informed about the purpose of the study, the therapeutic and adverse effects of DEX, and will provide informed consent. Each participant will be assigned a unique study number, and data will be collected and analysed in a de-identified but re-identifiable form.

### Recruitment

Participants will primarily be recruited through general practices, using flyers and direct GP referrals. Additional recruitment methods may include digital and social media advertisements, as well as articles in community newspapers.

Potential participants will first undergo a screening interview to assess their eligibility, followed by baseline investigations for those deemed likely to qualify. Once eligibility is confirmed, informed consent will be obtained, and participants will be randomised (see Fig. 1).

GPs with an interest in obesity management, and who are able to commit to the study requirements, will be recruited as study investigators. These GPs will be trained in the study protocol and procedures, with training provided by a member of the research team.

### Sample size

Sample size and power calculations for the primary analysis are centered on the expected weight loss at the 6-month endpoint. Based on the findings from Winslow et al., which indicated a significant weight reduction of 6.5 kg (95% CI: −10.0, −3.0) between treatment groups following 6 months of phentermine/topiramate therapy, we have designed our study to detect a similar effect [4]. To ensure adequate statistical power, we plan to recruit a total of 58 participants, with 29 assigned to each treatment arm. This sample size accounts for a projected attrition rate of 30% at 6 months, as observed in Poulton et al. [5]

The sample size calculation was based on 6-month outcomes, and that analyses at 2 years should be interpreted as exploratory and hypothesis-generating.

### Baseline investigations

a) Electrocardiography (ECG)
b) Echocardiogram (ECHO)
c) Blood biochemistry: Full Blood Count (FBC), Liver Function Tests (LFT), Electrolytes, Urea, and Creatinine (EUC), glucose, insulin, HbA1c, lipids, Thyroid-Stimulating Hormone (TSH), and high-sensitivity C-reactive protein (Hs-CRP)

### Baseline measures

At baseline, a comprehensive assessment will be conducted to gather key demographic information, including age, gender, and socio-economic status, as well as clinical data related to comorbidities such as hypertension, diabetes, cardiovascular disease, and sleep disorders. Additionally, psychosocial factors will be evaluated, including lifestyle behaviours, dietary patterns, physical activity levels, and mental health status, such as stress, depression, or anxiety, all of which could impact weight management outcomes. Anthropometric measurements will be recorded, including weight, height, BMI, and waist circumference.

Participants will also have an initial consultation with a dietician, during which personalised dietary recommendations and exercise plans will be provided, aligning with the overall goal of facilitating long-term weight loss. This consultation will serve as the foundation for the ongoing diet and exercise program throughout the trial.

Table 1 outlines the full range of data to be collected and their frequency of collection during the study. Regular monitoring of vital signs, assessment of adherence to diet and exercise programs, and weight will take place, alongside evaluations of psychotropic effects using standardised scales such as the Amphetamine Interview Rating Scale [6]. These parameters will be assessed at key intervals during the treatment phase. Adjustments will be made to study medication dosing and lifestyle intervention advice based on clinical grounds and questionnaire results to ensure patient safety and optimise outcomes.

**Table 1:** Schedule of Assessments and Data Collection. This table outlines the data collection schedule throughout the study, detailing the parameters recorded at baseline, during dose changes, maintenance, and after ceasing treatment.

	Baseline	Pre dose change	Maintenance	After ceasing
***Anthropometric data***				
*Weight*	X	X	X	X
*Height*	X			
*Waist circumference*	X			X
*Blood pressure*	X	X	X	X
*Heart rate*	X	X	X	X
***Demographic data***				
*Age*	X			
*Gender*	X			
*Enrolment questionnaire*	X			
*Other Medications*	X	X	X	X
***Investigations***				
*Echocardiogram*	X			X
*ECG*	X		X	
*Blood test (fasted)*	X			X
***Questionnaires***				
*PHQ-9 for depression*	X			X
*Amphetamine interview rating scale*	X	X	X	X
*Exercise and diet assessment form*	X	X	X	X

### Randomisation and blinding

Participants will be randomised to receive either immediate-release DEX tablets or a placebo, following a pre-determined randomisation sequence generated by a computer system. The allocation ratio will be 1:1, using a block size of 4. The DEX and placebo tablets will be identical in appearance and dispensed in containers that conceal the treatment allocation. Participants and investigators will remain blinded throughout the study. Unblinding will only occur if clinically necessary, such as in cases of severe adverse reactions or medical emergencies where knowledge of the treatment allocation is essential for patient safety. Blinding efficacy will be assessed at the end of the intervention using the Bang Blinding Index (BBI) for both participants and investigators to evaluate the success of treatment allocation concealment.

### Dose titration

Treatment will commence with either DEX or placebo tablets at a dose of 5 mg (1 tablet) twice daily. Participants will then undergo weekly incremental increases of 5 mg (1 tablet) twice daily as tolerated, up to a maximum dose limit of 30 mg (6 tablets) twice daily. Dosage adjustments will be primarily guided by any adverse effects experienced, which will be evaluated through patient history, the AIRS, and measurements of blood pressure (BP) and heart rate.

Down titration of the study medication dose will occur if a participant reports significant adverse effects such as increased anxiety, adverse mood changes, or severe insomnia. Additionally, if BP readings exceed 140/90 mmHg or heart rate increases beyond 100 beats per minute, this may also prompt a dose adjustment. Conversely, if participants demonstrate good tolerance with minimal side effects, stable BP readings within normal limits, and a heart rate under 100 beats per minute, the current dose may be maintained. In the final month of treatment, the dosage will be gradually reduced by 10 mg (2 tablets) each week to ensure a gradual tapering process.

### Study questionnaires

The Patient Health Questionnaire for Depression (PHQ-9) is a widely utilised and validated tool that assesses the severity of depression in both clinical and research settings [7]. This questionnaire evaluates each of the nine criteria outlined in the Diagnostic and Statistical Manual of Mental Disorders, Fourth Edition (DSM-IV), using a rating scale that ranges from 0 (not at all) to 3 (nearly every day). Administered at baseline, the PHQ-9 serves as a critical screening instrument for identifying active depressive disorders.

Similarly, the Ampetamine Interview Rating Scale (AIRS) is a validated scale employed throughout the treatment period, particularly during dose titration, to monitor participant responses. This scale comprises 34 questions with graded responses based on a Likert scale, covering various symptom categories, including much less than normal, less than normal, normal, more than normal, and much more than normal. The AIRS evaluates important factors such as hunger, mood, and sleep, with self-rated responses guiding dose adjustments as needed [6].

Additionally, the exercise-diet assessment form has been developed by the research team as a practical tool for tracking daily physical activity and dietary indiscretions. Participants complete this form weekly, and it is reviewed in consultations with their clinician. Moreover, it serves as an adherence record for researchers, ensuring that both dietary and exercise commitments are monitored effectively throughout the study.

### Outcome measures

Outcome measures will be collected at baseline, during each dose adjustment visit, during the maintenance phase of treatment, and following cessation of medication at 1 week, and at 1, 3, 6, 12, 18, and 24 months, as illustrated in Fig. 1.

Primary Outcome

• Change in Body Weight: Change in body weight from baseline measured at 6 months during treatment and at 2 years following discontinuation of study medication.

Secondary Outcomes

1. Cardiovascular Outcomes: Changes in cardiovascular parameters and the incidence of adverse cardiovascular events.
2. Baseline Mental Health Assessment: Identification of possible undiagnosed active depressive disorders at baseline.
3. Neuropsychiatric Effects: Changes in mood, cognition, and physiological responses during treatment as measured by the Amphetamine Interview Rating Scale (AIRS).
4. Feasibility will be assessed by recruitment and retention rates, treatment adherence, ability to achieve target dosing, and the frequency of adverse events or treatment discontinuation.

### Data collection

Data will be collected prospectively at predefined study time points. Assessments will occur at baseline, during each dose adjustment visit, throughout the maintenance phase, and following cessation of medication at 1 week and at 1, 3, 6, 12, 18, and 24 months, as outlined in Fig. 1.

At baseline, demographic data, medical history, current medications, and relevant clinical characteristics will be recorded. Baseline clinical assessments will include body weight, cardiovascular parameters (including ECG, echocardiogram, blood pressure and heart rate), and screening for possible undiagnosed depressive disorders.

During treatment and follow-up visits, body weight and cardiovascular parameters will be measured and recorded. Any adverse events, including cardiovascular events, will be documented throughout the study period. While participants are receiving the study medication, mood, cognition, and physiological responses will be assessed using the Amphetamine Interview Rating Scale (AIRS).

All data will be collected during routine general practice consultations by the treating clinician or trained study personnel and recorded in a secure electronic study database. Data completeness and accuracy will be monitored regularly, and any discrepancies will be reviewed and resolved where possible.

Participant information will be de-identified prior to analysis and stored securely in accordance with institutional data governance and privacy regulations.

### Data analysis

The primary analysis will adopt an intention-to-treat approach to ensure that all randomised participants are included in the evaluation, regardless of adherence to the treatment protocol. The primary outcome, the change in weight from baseline to both 6 months and 2 years, will be assessed using simple and multiple linear regression, adjusting for prespecified covariates such as age, sex, baseline BMI, and comorbidities. Secondary outcomes, including cardiovascular parameters, mood and cognition (AIRS scores), and feasibility metrics, will be analysed using appropriate regression models or repeated-measures mixed-effects models for longitudinal data. Missing data will be addressed primarily using available data under intention to treat; where missingness exceeds 10–15%, multiple imputation and complete-case analyses will be conducted as sensitivity analyses. Exploratory subgroup analyses by sex, baseline BMI, or baseline depressive status may also be performed. Adverse events will be summarised descriptively by type, severity, and relationship to treatment, with serious events reported individually.

### Indications for early withdrawal

Participants may withdraw or be withdrawn from the study prematurely under specific circumstances to ensure their safety and the integrity of the research. The following criteria will guide decisions regarding early withdrawal:

a) Significant Adverse Event (SAE): If a participant experiences a SAE that is deemed related to the intervention, they will be promptly withdrawn from the study. SAEs may include, but are not limited to, severe cardiovascular issues, psychiatric disturbances, or any other health complications that arise during the treatment period. The safety of participants is paramount, and frequent monitoring will be conducted to identify and address any adverse effects promptly. Recognised medication side effects, such as dry mouth, insomnia or mood changes will be discussed with the participant and managed according to participant toleration. Management may include a dose reduction.
b) Withdrawal of Consent: If a participant chooses to withdraw, their decision will be respected. The participant will be invited to give the reason for withdrawal.

Through these early withdrawal criteria, we aim to uphold participant safety and maintain the quality and reliability of the study data. Any participant withdrawn from the study will be offered appropriate follow-up care and support as needed.

### Optimising attendance and handling missing data

To minimise missed follow-up assessments, all participants will be contacted ahead of their scheduled visits to confirm their attendance. If necessary, assessments can be postponed or rescheduled, with appropriate documentation maintained. Attendance at assessments during the dose titration phase is mandatory before considering any dose increases. Additionally, we will strive to collect follow-up data from participants who are unable to attend appointments during the maintenance dose phase or after treatment cessation by telephone. Participants will be asked to provide measurements taken from personal weight and blood pressure measuring equipment.

While we are committed to optimising adherence, some missing data are anticipated. To address this, we will compare baseline characteristics between participants with and without assessments at 6 and 24 months to identify potential biases in our final analysis. We will also investigate the reasons for study dropouts to better understand the mechanisms behind missing data, including any adherence issues related to the study protocol. Data will be reviewed regularly to identify missing or inconsistent entries, and where possible, clarification will be sought from the treating clinician or study records.

For the primary analysis, available data will be analysed using appropriate statistical methods depending on the extent and pattern of missingness. If the proportion of missing data is low, analyses will be conducted using complete case analysis. If missing data are substantial, appropriate statistical techniques such as multiple imputation will be considered to reduce potential bias.

Sensitivity analyses may be conducted to assess the impact of missing data on the primary outcome.

### Reproducibility of study procedures

A comprehensive procedure manual has been created to standardise all aspects of the study, including patient recruitment, measurement assessment, data entry, management, analysis, and security. All study staff will receive training tailored to their specific roles. The manual includes detailed monitoring plans designed to safeguard patient welfare and ensure data integrity.

### Protocol validation

Poulton et al., treated 14 people with obesity with DEX for 6 months [5]. There was a mean weight loss of 10.6 ± 8.4 kg, (P < 0.001), with 7 participants (50%) losing >10% of their total body weight. Six months post DEX treatment, weight regain reduced the mean weight loss to 7.0 ± 10.6 kg (p = 0.035). However, 3 participants still recorded a weight reduction of >10% and 4–5 participants recorded a weight reduction between 5–10% of their baseline body weight. Other important clinical outcomes identified a reduction in the severity of obstructive sleep apnoea in one participant, a decrease in the amount of insulin required for diabetes control in another and a reduction in the number of antihypertensive medications in a third participant.

## Limitations

None.

## CRediT Author statement

**Natalie Gauci:** Conceptualization, methodology, writing – original draft, project administration. **Emily Hibbert:** Conceptualization, methodology, writing- review and editing, supervision. **Hazer Khalifa:** Project administration. **Alison Poulton:** Conceptualization, methodology, writing- review and editing, supervision.

## Related research article

None.

## Declaration of competing interest

The authors declare the following financial interests/personal relationships which may be considered as potential competing interests:

NG and EH have no competing interests. KH is an employee of Eli Lilly. AP discloses personal fees and non-financial support from Shire/Takeda, outside the submitted work; and book royalties from Disruptive Publishing (ADHD Made Simple).

## Data Availability

No data was used for the research described in the article.
